# Multi-Disciplinary Care of Hilar Cholangiocarcinoma: Review of Guidelines and Recent Advancements

**DOI:** 10.3390/cancers16010030

**Published:** 2023-12-20

**Authors:** Vennila Padmanaban, Samantha M. Ruff, Timothy M. Pawlik

**Affiliations:** Department of Surgery, Division of Surgical Oncology, The Ohio State University Wexner Medical Center and James Comprehensive Cancer Center, Columbus, OH 43210, USA; vennila.padmanaban@osumc.edu (V.P.);

**Keywords:** extrahepatic cholangiocarcinoma, targeted therapy, hilar cholangiocarcinoma, surgery

## Abstract

**Simple Summary:**

Upfront surgery with adjuvant capecitabine is the only curative treatment for hilar cholangiocarcinoma. Unfortunately, most patients do not present with resectable disease and are treated with a combination of locoregional therapy and systemic therapy. This review will summarize existing literature and ongoing clinical trials focused on the multidisciplinary care of hilar cholangiocarcinoma.

**Abstract:**

Cholangiocarcinoma (CCA) is a rare malignancy of the intrahepatic and extrahepatic biliary ducts. CCA is primarily defined by its anatomic location: intrahepatic cholangiocarcinoma versus extrahepatic cholangiocarcinoma. Hilar cholangiocarcinoma (HC) is a subtype of extrahepatic cholangiocarcinoma that arises from the common hepatic bile duct and can extend to the right and/or left hepatic bile ducts. Upfront surgery with adjuvant capecitabine is the standard of care for patients who present with early disease and the only curative therapy. Unfortunately, most patients present with locally advanced or metastatic disease and must rely on systemic therapy as their primary treatment. However, even with current systemic therapy, survival is still poor. As such, research is focused on developing targeted therapies and multimodal strategies to improve overall prognosis. This review discusses the work-up and management of HC focused on the most up-to-date literature and ongoing clinical trials.

## 1. Introduction

Cholangiocarcinoma (CCA) is a rare and aggressive cancer that arises from the biliary ducts and is defined by its anatomic location: intrahepatic or extrahepatic. Extrahepatic CCA can be further separated into hilar cholangiocarcinoma (HC) or distal CCA. HC arises from the common hepatic duct, while distal CCAs are tumors that arise from the common bile duct below the cystic duct insertion. Increasing evidence suggests that intrahepatic and extrahepatic CCA are biologically different cancers and should have individualized treatment strategies. However, due to the rarity of these cancers, substantive experience is limited to a few specialized centers and it can be difficult to efficiently accrue patients for clinical trials to advance the field [1,2,3,4]. For patients with resectable disease, the standard of care is upfront surgery with adjuvant capecitabine. While surgery confers the greatest survival advantage, most patients will develop recurrent or metastatic disease afterwards [2,5,6]. As such, it is critical to select patients for surgery who will derive the greatest oncologic benefit while balancing the morbidity and mortality associated with these complex operations. Furthermore, more than one-half of patients with HC already have locally advanced or metastatic disease at the time of diagnosis. Systemic therapy options for these patients are limited and do little to improve long-term survival [7,8,9].

Given the tumor heterogeneity associated with CCA, research has focused on developing effective targeted therapies [4]. Targeted therapy carries a better side effect profile than cytotoxic chemotherapy and can be personalized based on the tumor’s genomic landscape. As such, molecular profiling should be performed in patients with advanced HC and patients should be referred to clinical trials as appropriate [10,11]. We herein review the work-up and management of HC focused on the most up-to-date literature and ongoing clinical trials.

## 2. Methods

A contemporary review using Pub Med, Science Direct, and clinicaltrials.gov was conducted to identify clinical trials, guidelines, and translational studies. The search was conducted in September 2023. The search strategy involved the key words “biliary tract cancer”, “cholangiocarcinomas”, “hilar cholangiocarcinoma”, “immunotherapy”, and “targeted therapy”, as well as individual genes and therapy names. Studies between 2000 and 2023 were included. Articles were identified and reviewed; significant findings and recent developments in therapeutics for hilar cholangiocarcinoma were collated and summarized.

## 3. Classification, Epidemiology and Risk Factors

**Figure 1:**
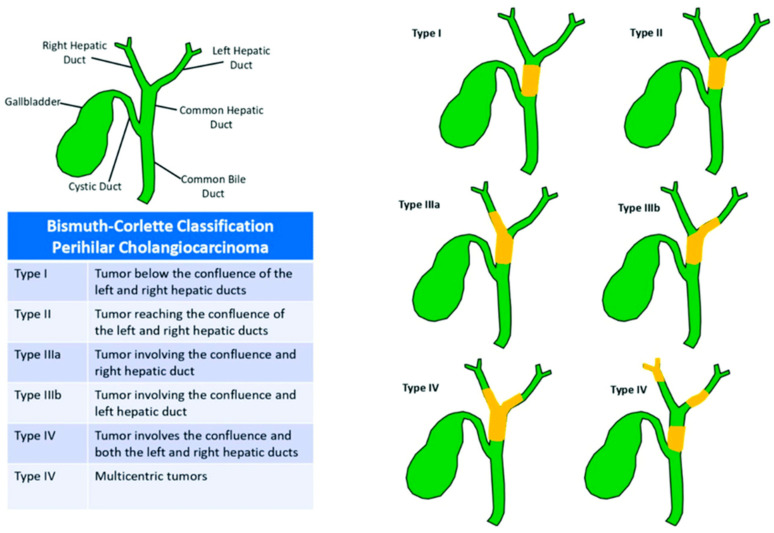
Bismuth–Corlette Classification of Perihilar Cholangiocarcinoma. This figure was re-printed with copyright permission from reference [17].

Cholangiocarcinoma (CCA) is a rare and aggressive tumor arising from the epithelial cells of the intra or extrahepatic biliary ducts [12]. There is tremendous heterogeneity among CCAs at the genomic, epigenetic, and molecular level, suggesting that intrahepatic (ICC) and extrahepatic (ECC) cholangiocarcinomas are biologically distinct cancers [1]. The incidence of CCAs (0.3–6 per 100,000) and mortality have been rising worldwide, particularly in South Asian countries including South Korea, China, and Thailand [1,13,14]. ECC can be divided anatomically into hilar cholangiocarcinoma (HC) arising from the common hepatic duct and distal CCA arising from the common bile duct below the cystic duct insertion. In addition to the common hepatic duct, HC can involve the right hepatic duct, the left hepatic duct, and/or the confluence [2,12]. HC represents 50–60% of all cholangiocarcinomas. The European Society for Medical Oncology (ESMO), the National Comprehensive Cancer Network (NCCN), and the Americas Hepato-Pancreato-Biliary Association (AHPBA) guidelines further define HC based on its extent within the biliary tree with the Bismuth–Corlette classification (Figure 1) [4,15]. The extent of HC disease guides treatment and determines resectability.

The majority of HC tumors are sporadic, with an unknown etiology [3]. Risk factors include choledochal cysts, choledocholithiasis or Caroli’s disease, cigarette smoking, alcohol, and systemic diseases like primary sclerosing cholangitis (PSC), cirrhosis, or chronic hepatitis B and C. Additionally, some studies suggest a relationship between the rising incidence of type II diabetes mellitus, cirrhosis, and alcoholic liver disease with increasing incidence of CCA [1,16]. The preponderance of HC in South Asia may be attributable to liver fluke infection [14].

## 4. Clinical Presentation and Pathology

Patients commonly present with cachexia, fatigue, and/or painless jaundice secondary to biliary duct obstruction. Unfortunately, painless jaundice at presentation often represents locally advanced or metastatic disease [18,19,20,21]. About 10% of patients will have concomitant cholangitis at presentation.

On pathology, HC may have invasive spread, showing more neural, perineural, and lymphatic involvement and subepithelial extension than intrahepatic cholangiocarcinomas [3,22]. There are three pathologic subtypes of ECC which utilize sclerosing (>70%), nodular (20%), and papillary (5–10%). Most mucinous adenocarcinomas are of the sclerosing and nodular subtypes, and may involve the biliary lymphatic plexus and progressively develop into a mass [2,19]. The majority of HCs express cytokeratin (CK)-7 and CK-20 [23].

Multistep carcinogenesis of biliary tumors may occur from the transformation of precursor pancreaticobiliary lesions in large ducts such as biliary intraepithelial neoplasm (BilIN) and intraductal papillary neoplasm of the bile duct (IPNB). These lesions are distinguished by size and pathologic proliferation patterns [22]. In particular, BilN may represent the most common precursor of HC, as well as large-duct intrahepatic CCA [20]. The analysis of BilN has helped clarify the carcinogenesis of biliary duct cancers, specifically identifying a high propensity of KRAS mutations (33%) [24,25]. Another precursor lesion, IPNB, is an analogue of the pancreatic intraductal papillary mucinous neoplasm. IPNBs are characterized into low, intermediate, and high-grade lesions based on cytology and architecture. As with HC histologic subtypes, IPNBs contain a component of invasive carcinoma or mucinous adenocarcinoma with potential for malignancy. As demonstrated in pancreatic adenocarcinoma, the loss of tumor suppressor SMAD4/DPC4 has been identified in BilN and IPNB precursor lesions and has been implicated in carcinogenesis. Surgical resection remains the mainstay of treatment, and invasive tumors arising in the setting of IPNB have a better prognosis [24].

The identification of precursor lesions varies among patients with intra- and extra-hepatic cholangiocarcinoma and represents an area of future diagnostic and therapeutic research [22,26]. The immunophenotypes of HC are more similar to those of pancreatic ductal adenocarcinoma, while intrahepatic cholangiocarcinoma and hepatocellular carcinoma share a resemblance [22]. Immunohistochemical studies have demonstrated unique phenotypes and progenitor cells that differentiate types of biliary cancers according to their anatomic sites of origin. The pathways of molecular signaling are a focus of investigation relative to their role in tumor growth and treatment resistance and represent areas for future therapeutic targets [24].

## 5. Diagnostic Testing and Staging

At initial presentation, bloodwork including a basic metabolic panel, coagulation studies, complete blood count, hepatitis panel, and hepatic function panel (alkaline phosphatase (ALP), gamma glutamyltransferase (GTT), bilirubin, and liver enzymes (AST/ALT)) should be obtained to evaluate for underlying liver dysfunction (Table 1) [27]. If suspected, then the patient should be referred to a hepatologist. While there are no tumor markers specific to CCA, carbohydrate antigen 19-9 (CA 19-9) should be checked, since it can be elevated in patients who produce Lewis antigen. However, hyperbilirubinemia can result in a falsely elevated CA 19-9. As such, patients should have this lab drawn after intervention for obstructive jaundice when the bilirubin has normalized. In patients without primary sclerosing cholangitis, CA 19-9 > 100 mg/dL has a sensitivity of 53% and a specificity of 75–90% for CCA [20].

Cross-sectional imaging with high-resolution contrast-enhanced computed tomography (CT) or magnetic resonance imaging/magnetic resonance cholangiopancreatography (MRCP) is critical to diagnosis and operative planning [2,3,4]. The MRCP should optimally be performed before biliary decompression so that the stent does not cause artifact and interfere with the images. These imaging modalities will also be used to assess for treatment response and eventual surveillance after resection [1]. Endoscopic retrograde cholangiopancreatography (ERCP) is performed with diagnostic and therapeutic intent. During ERCP, a stent is placed to relieve the biliary obstruction and then biliary brushings are collected to review for malignant cells. Additionally, at the time of ERCP, patients should undergo an endoscopic ultrasound (EUS) to evaluate for lymphadenopathy or a mass. If either are present, a fine needle aspiration can be performed, and tissue sent to pathology; this tissue can be used for histologic classification and next-generation sequencing [23]. The proximal and distal extent of HC can be difficult to ascertain from imaging alone. As such, the combination of ERCP, EUS, and SpyGlass cholangioscopy may help define the extent of the disease. Among patients who cannot undergo ERCP, an external biliary drain can be placed by percutaneous transhepatic cholangiogram (PTC) to divert the bile. In combination with transhepatic drainage, a transgastric EUS-guided rendezvous procedure has recently gained popularity to help stent across the malignant stricture [28]. A CT scan of the chest should be performed to complete staging [10]. HC is not fludeoxyglucose (FDG)-avid, and therefore positron emission tomography (PET) evaluation is a low-sensitivity modality and is not recommended [2,19].

## 6. Principles of Surgical Resection

**Figure 2:**
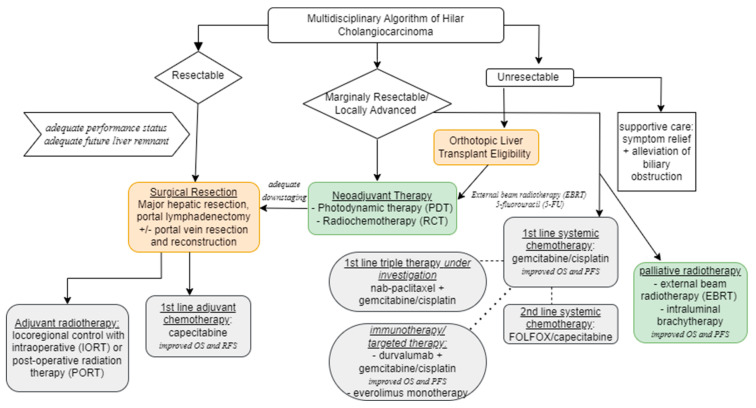
Multidisciplinary Algorithm of Hilar Cholangiocarcinoma (OS = overall survival; PFS = progression-free survival).

Patients with resectable HC and no metastatic disease should undergo upfront surgery, and surgery remains the cornerstone of treatment. However, resection may not be feasible due to technical, biologic, and/or patient-specific factors (Table 2) [29]. As noted by the APHBA consensus statement, contraindication for resection in patients with HC includes (1) bilateral segmental duct extension (2) unilateral atrophy with either contralateral segmental duct or vascular inflow involvement, and (3) unilateral segmental duct extension with contralateral vascular inflow involvement [19]. The decision for surgery should be made in collaboration with a multidisciplinary tumor board in the context of other treatment modalities, which are summarized in Figure 2.

Given the anatomic location of HC, patients often require a bile duct resection, partial hepatectomy, and biliary enteric reconstruction [31]. NCCN guidelines also recommend caudate lobe resection to improve obtaining a negative margin [10]. The extent of resection is based on the Bismuth–Corlette classification system (Figure 1). In general, type 1, 2, and 3a tumors are treated with extended right hepatectomy. Type 3b lesions are resected with a left hepatectomy. Historically, patients with type 4 tumors were considered unresectable due to bilateral involvement of the biliary tree. However, recent studies exploring resection for these patients have demonstrated improved survival [32,33].

These complex operations carry an elevated morbidity and mortality risk. Prior to surgery, patients’ overall fitness and co-morbidities must be assessed to ensure that they can tolerate a resection. The use of prehabilitation and rehabilitation exercise programs is recommended to optimize patient performance status [31]. Patients with ECOG (Eastern Cooperative Oncology Group) score ≥ 3 are unlikely to benefit from treatment and should be managed with best supportive care [1]. Additionally, liver volumetric analysis should be performed to ensure an adequate future liver remnant (FLR) to prevent postoperative hepatic insufficiency. Patients with healthy livers need an FLR of at least 30%, while individuals with cirrhosis need an FLR of at least 40% [34,35,36]. Chemotherapy can also cause imperceptible damage to the liver and may require an FLR of around 40% when feasible. For patients with potentially small FLR, portal vein embolization can be performed to accelerate hypertrophy of the FLR. Hyperbilirubinemia can cause hepatic dysfunction; therefore, even when asymptomatic, some patients require biliary drainage of the FLR prior to surgery [30,31,37].

Unfortunately, given the aggressive nature of HC, 50–70% of patients will develop recurrence after surgery [5,18]. Achieving an R0 resection can prevent early local recurrence and improve survival [8]. Intraoperative frozen examination of the bile duct margins should be performed. If proximal or distal margins are positive on frozen pathology, then further resection should be performed. In rare situations, patients may have a positive margin on frozen pathology, but be unable to undergo further resection of the bile duct. Additionally, it is important to ensure that the radial margin in the porta hepatis and hepatic parenchymal transection line is grossly negative for disease [31]. On cross-sectional imaging, portal vein involvement may be evident from occlusion or irregularity of the vein contour, or obstruction of flow. Radiographic findings may include hepatic lobar atrophy of the involved side and contralateral lobar hypertrophy. Highly selected patients may benefit from portal vein resection/reconstruction to achieve a negative margin and improve local recurrence rate.

Several clinical and prognostic factors are associated with improved survival. In a 2020 retrospective analysis of patients with HC who underwent curative intent surgery, lymph node metastasis (LNM) was the only significant prognostic factor associated with both worse overall survival (OS) and disease-free survival (DFS) [38]. The presence of locoregional LNM (N stage), higher tumor invasion depth (T stage), and higher American Society of Anesthesiologists (ASA) score were associated with worse OS [38]. The presence of LNM carries important prognostic information. As such, portal lymphadenectomy with at least six lymph nodes harvested is the current standard of care [10].

## 7. Orthotopic Liver Transplantation

The European Network for the Study of Cholangiocarcinoma (ENS-CC), the NCCN, and the ESMO guidelines recommend neoadjuvant chemoradiotherapy and liver transplant evaluation for patients with unresectable HC or HC in the setting of PSC [1,13,39]. The neoadjuvant therapy regimen in this setting includes external beam radiotherapy (EBRT) and an infusion of 5-fluorouracil (5-FU) [19].

The Organ Procurement and Transplant Network (OPTN) guidelines require patients to have cholangiography with evidence of a malignant-appearing stricture with concomitant (1) biopsy or cytology-proven malignancy, (2) carbohydrate antigen 19-9 greater than 100 U/mL and the absence of cholangitis, (3) aneuploidy, or (4) hilar mass that is <3 cm in radial diameter. Additionally, the tumor must be considered unresectable due to anatomic technical considerations or underlying liver disease. Operative staging must be performed to exclude patients with regional hepatic LNM, the hilar mass must be <3 cm, and patients must demonstrate disease stability during neoadjuvant therapy [40,41,42]. Patients considered for transplant candidacy may not undergo transperitoneal needle aspiration for biopsy due to the risk of tumor seeding and dissemination [43].

Although early initial studies demonstrated a high recurrence rate of 55%, further multicenter retrospective studies demonstrated a 5-year DFS of 65% as well as a 5-year intention-to-treat survival of 53% [1,44]. The 5-year survival rate in patients with PSC who have undergone liver transplantation with neoadjuvant chemoradiotherapy is more variable, between 55–82% [19,40].

## 8. Locoregional Therapy

### 8.1. Neoadjuvant Radiochemotherapy (RCT) in Resectable Cancer

Neoadjuvant therapy can improve the rate of margin-negative resection, downstage patients initially thought to be unresectable, treat micrometastatic disease, and improve patient selection for major surgery. Patients who progress quickly on neoadjuvant therapy represent those with poor disease biology who will not derive an oncologic benefit from these surgeries that carry an elevated risk of morbidity and mortality. There are limited studies investigating the use of radiochemotherapy (RCT) in the neoadjuvant setting for locally advanced HC. The use of RCT was initially described in 1997 by McMasters et al. in a prospective study that treated patients with ECC with neoadjuvant 5-FU and EBRT [45]. Although the study size was limited, 44% of patients were able to undergo an operation. The rate of margin-negative resection was 100% for the preoperative chemoradiation group versus 54% for the surgery upfront cohort [45]. In 2018, a Japanese retrospective study demonstrated that neoadjuvant chemotherapy successfully downstaged 75% of their patients from unresectable to resectable disease [46]. Studies for neoadjuvant radiochemotherapy for resectable cancer are limited to retrospective studies and case reports [47].

### 8.2. Neoadjuvant Photodynamic Therapy

Photodynamic therapy (PDT) is a clinically approved procedure that relies on cytotoxic activity, directed tumor cell death, and induction of a local inflammatory reaction. PDT is a minimally invasive technique as it is administered through either ERCP or percutaneous transhepatic cholangiocatheter (PTC) with biliary cholangiography. A cylindrical optical fiber is introduced to the site of tumor stenosis [48,49]. A photosensitizing drug that accumulates in tumor cells is administered and a laser light is administered to the malignant cells. The activated photosensitizer forms oxygen radicals that damage tumor cells [48]. PDT can be performed in outpatient and ambulatory settings, with adverse effects including skin photosensitization and ERCP- or PTC-related complications (e.g., acute cholangitis, pancreatitis, hematobilia, and liver abscess). A small phase II pilot study demonstrated an R0 resection in all seven patients treated with neoadjuvant PDT prior to resection, with a 1-year recurrence-free survival rate of 83% [50]. There is a need for additional data to support curative effects [48,49].

### 8.3. Radiation in the Adjuvant and Palliative Setting

No studies have demonstrated a benefit from postoperative radiation after R0 resection [20]. However, several studies have demonstrated the benefit of adjuvant chemoradiation in resected HC with positive margins. Todoroki et al. reported results in 63 patients with resected HC and R1 margins [11,51]. The authors demonstrated improved locoregional control in the adjuvant therapy group compared to the resection-alone group (80% versus 31%, respectively). Patients were treated with intraoperative radiotherapy (IORT) and/or postoperative radiotherapy (PORT) [51].

The use of radiation therapy in the palliative setting has been well-supported in patients who are not candidates for resection or who have undergone palliative bypass. The two most commonly used radiation therapies are EBRT and intraluminal brachytherapy delivered by endoscopic or percutaneous approaches. Studies with palliative radiation therapy demonstrate a survival range of 9–14 months [20]. A 2015 prospective study examined the use of stereotactic body radiotherapy (SBRT) among patients with localized, non-metastatic intrahepatic and hilar cholangiocarcinoma and demonstrated a median overall survival of 17 months as well as a median progression-free survival of 10 months [52].

## 9. Systemic Therapy

### 9.1. Adjuvant Chemotherapy

Due to the rarity of HC, clinical trials can be difficult to implement. As such, most studies treat all biliary tract cancers (BTC) together (gallbladder, ECC, and ICC). The recommendations of various society guidelines are summarized in Table 3. However, these cancers have unique underlying biology, and therefore, interpreting the results of these trials can be difficult [53]. The BCAT (Bile Duct Cancer Adjuvant Trial) study was a randomized phase III trial conducted in 48 centers in Japan that evaluated the use of gemcitabine versus observation in patients with resected HC or distal CCA. Unfortunately, the trial failed to demonstrate a significant survival advantage with the addition of gemcitabine [53]. In the landmark phase III BILCAP trial, patients with resected BTCs were randomized to observation or adjuvant capecitabine [53,54]. This study demonstrated a survival advantage in the adjuvant capecitabine cohort compared to the observation cohort (median OS: 51 months versus 36 months, respectively). Of note, there was an improvement in median recurrence-free survival after treatment (median RFS—capecitabine cohort: 25 months versus observation cohort: 18 months). In turn, capecitabine has been generally accepted as the standard-of-care adjuvant therapy for patients with a resected BTC [53,54].

### 9.2. Systemic Chemotherapy for Locally Advanced or Metastatic HC

Based on the ABC-02 (Advanced Biliary Cancer) trial, the recommended first-line chemotherapy for locally advanced or metastatic BTC is a combination of gemcitabine and cisplatin. This study extended the initial findings of the phase II trial, ABC-01, which showed an improvement in 6-month progression-free survival from 47.7% to 57.1% in patients who received a combination gemcitabine and cisplatin [55]. ABC-02 demonstrated a median overall survival of 11.7 months among the 204 patients in the cisplatin and gemcitabine cohort and 8.1 months among the 206 patients in the gemcitabine-alone cohort (HR 0.64) [7]. Improved survival was demonstrated in the HC subgroup of 57 patients (hazard ratio 0.59).

Based on promising results in a phase II trial, the Southwestern Oncology Group (SWOG) 1815 trial is currently evaluating the addition of nab-paclitaxel to gemcitabine and cisplatin in patients with advanced BTC. In theory, nab-paclitaxel will deplete the stromal-rich environment of cholangiocarcinoma and allow for improved delivery of gemcitabine and cisplatin. Based on preliminary results, the addition of nab-paclitaxel did not statistically improve median OS, but further analysis is ongoing to see if a subset of patients with BTC may benefit [56]. Additional investigations are ongoing to evaluate the role of triple therapy combinations in the first-line setting, such as S1 (tegafur, gimeracil and oteracil) or FOLFIRINOX (5-FU, oxaliplatin and irinotecan) [5,13].

FOLFOX and capecitabine are the recommended second-like chemotherapies that have been demonstrated to confer a survival benefit in patients with CCA [57]. The phase III ABC-06 clinical trial administered FOLFOX to patients with BTCs who progressed on first-line cisplatin-gemcitabine. There was a clinically meaningful 6- (35.5% versus 50.6%) and 12-month (11.4% versus 25.9%) survival difference [58].

### 9.3. Immunotherapy

The immune system plays a critical role in cancer treatment. Cancer cells can upregulate expression of inhibitory ligands that bind to receptors on T cells and cause immunosuppression. Through these immune checkpoints, cancer cells evade the immune system, leading to carcinogenesis and disease progression. The most commonly targeted immune checkpoints are programmed cell death ligand 1 (PDL-1) and cytokine T-lymphocyte associated protein 4 (CTLA-4). While chemotherapy remains the mainstay of systemic treatment for cholangiocarcinoma, recent studies have demonstrated promising results in the use of immune checkpoint inhibitors (ICIs) in combination with systemic chemotherapy [59]. The use of pembrolizumab was evaluated in the KEYNOTE-028 and KEYNOTE-158 trials in patients with PD-L1 expression on tumor biopsy. This study demonstrated anti-tumor activity in 6–13% of patients and manageable toxicities. These early-phase studies lack specific information regarding tumor location (e.g., ICC versus ECC) [59,60].

**Figure 3:**
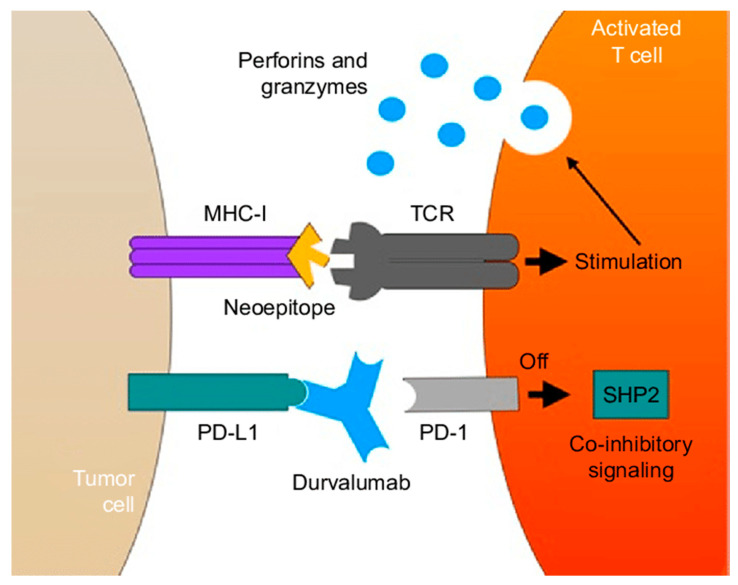
Mechanism of durvalumab antibody which blocks PD-1 and PD-L1 interaction to prevent a SHP2-mediated co-inhibitory signal. The neoepitope MHC-1 acts as a signal in stimulating an immune response and leading to destruction of the tumor cell. Abbreviations: PD-1, programmed cell death-1; PD-L1, programmed cell death ligand-1; SHP2, Src homology 2 domain-containing protein tyrosine phosphatase; MHC, major histo compatibility complex; TCR, T-cell receptor. This figure was re-printed with copyright permission from reference [62].

The recent Topaz I phase III trial evaluated the combination of immunotherapy and chemotherapy in patients with advanced BTCs. The addition of durvalumab, a PD-L1 inhibitor, to gemcitabine and cisplatin significantly improved survival without additional toxicity (Figure 3). The durvalumab cohort had a median OS and PFS of 12.8 months and 7.2 months, respectively, compared to 11.5 months and 5.7 months, respectively, in the placebo cohort. As such, durvalumab in combination with gemcitabine and cisplatin is recommended as the standard of care for patients with advanced BTCs [61]. Research is currently ongoing and focused on other combinations of immunotherapy and chemotherapy [17,59].

### 9.4. Targeted Therapy

While all cholangiocarcinoma subtypes share several somatic mutations, including TP53 and KRAS, recent next-generation sequencing has identified unique genetic aberrations dependent on the CCA subtype [62]. While most sequencing studies group HC and distal CCA together for analysis, one study suggested that HC has a distinct molecular subtype [63]. High-frequency HC subtype-specific mutations include TP53, KRAS, mTOR, ABL1, NOTCH1, PBRM1, PIK3CA, ARID1A, NF1, and EGFR [64]. The prevalence of EGFR mutations supports future investigation of tyrosine kinase inhibitors as a potential therapeutic option. Other genes with a lower prevalence of mutations included ATM, CHEK1, BRCA1, and BRCA2. Targetable mutations in HC were recently identified in MAP3K9, similar to lung cancer [64]. Mutations in mTOR and ABL1 may contribute to oncogenesis in HC. In a phase II study, everolimus, an mTOR inhibitor, demonstrated some clinical activity as a monotherapy in patients with advanced BTCs [65]. Current evidence regarding targeted therapy for HC is limited and more work in the pre-clinical setting is necessary to isolate targetable genetic aberrations [59,66].

## 10. Conclusions and Future Directions

Hilar cholangiocarcinoma is a subtype of CCA that arises from the common hepatic duct and extends proximally into the hepatic duct confluence, the right hepatic duct, and/or the left hepatic duct. When feasible, HC should be managed with upfront surgery followed by adjuvant capecitabine. Unfortunately, most patients develop recurrent or metastatic disease after resection and many will present with advanced disease that is unresectable. As such, the focus has shifted to improving systemic therapy options and neoadjuvant radiation and chemotherapy to downstage patients for a resection. Despite significant advances in immunotherapy and identifying targetable genetic aberrations, we have barely scratched the surface of what there is to learn about treating HC. Continued translational research with collaboration between the lab and clinical teams will be critical to moving the field forward. Additional large and prospective multicenter trials to facilitate quick accrual to test these therapies are needed.

## Figures and Tables

**Table 1:** Recommended Diagnostic Modalities for Hilar Cholangiocarcinoma.

Blood Tests	Imaging	Endoscopy	Tissue Sampling
- basic metabolic panel - hepatitis panel - hepatic function panel - complete blood count - coagulation studies	- high-resolution abdominal CT - staging CT chest - MRI - MRCP	- EUS - intraductal ultrasound - cholangioscopy	- aspiration - brush cytology - biopsy

**Table 2:** Criteria for Unresectability of Hilar Cholangiocarcinoma [30].

Local invasion - bilateral intrahepatic duct spread to secondary biliary radicals - involvement of the main trunk of the portal vein - bilobar involvement of hepatic arterial or portal venous branches - unilateral hepatic arterial involvement with evidence of contralateral duct spread
Inadequate future liver remnant - <30% FLR in normal hepatic parenchyma - Inadequate portal venous and hepatic arterial inflow, hepatic venous drainage and biliary drainage Hepatic cirrhosis
Distant metastatic disease - Hepatic metastases - Lymph node metastases beyond portal vein, hepatic artery, celiac axis and peripancreatic distribution
Medically unfit patients unable to tolerate a major hepatic resection

**Table 3:** Recommended Treatments for Hilar Cholangiocarcinoma Among Society Guidelines.

**Recommended Treatments**	**ESMO** European Society for Medical Oncology	**ENS-CCA** European Network for the Study of Cholangiocarcinoma	**NCCN** National Comprehensive Cancer Network	**AHPBA** Americas Hepato-Pancreato-Biliary Association	**ASCO** American Society of Clinical Oncology
**Neoadjuvant Treatment**	- durvalumab + gemcitabine-cisplatin recommended to downstage locally advanced cancers	- recommended in setting of transplantation	- no preferred treatments recommended	- recommending in setting of transplantation	*Guidelines address adjuvant treatments for resected malignancies*
**Adjuvant/Locoregional Therapies**	- capecitabine - adjuvant radiation considered in limited cases after adjuvant capecitabine in select patients (R1 resection)	- transarterial chemoembolization (TACE) - intraductal radiofrequency ablation (RFA)	- capecitabine - fluoropyridine-based chemoradiotherapy	- chemoradiation recommended for node positive and margin positive disease - role of adjuvant chemotherapy not clearly defined	- capecitabine moderate recommendation for chemoradiation - chemoradiotherapy (R1 resection)
**Primary Systemic Treatment of Unresectable/Metastatic Disease + Immunotherapy**	- durvalumab + gemcitabine-cisplatin - photodynamic therapy(PDT) and intraductal radiofrequency ablation are considered investigational	- Gemcitabine-cisplatin - Capecitabine - Gemcitabine-oxaliplatin - mFOLFOX - Fluorouracil-cisplatin *No recommendations for immunotherapy, citing need for additional research*	- durvalumab + gemcitabine-cisplatin - pembrolizumab + gemcitabine + cisplatin - PDT	- gemcitabine + cisplatin - recommend definitive chemoradiation	*Guidelines address adjuvant treatments for resected malignancies*
**Targeted Therapy**	- molecular analysis recommended during first line-therapy for advanced disease - ivosidenib (IDH1 mutations), FGFR inhibitors, pemrolizumab (MSI-H/dMMR), dabrafenib-trametinib (BRAF mutations) recommended, awaiting European Medicines Agency approval	- recommend additional clinical trials; no or only very modest surviual benefits	- useful in certain circumstances - pemrolizumab (panel states that data is limit) - dabrafenib and trametinib - comprehensive molecular profiling recommended in unresectable/metastatic disease	*Not commented in guidelines*	*Guidelines address adjuvant treatments for resected malignancies*
**Transplantation**	neoadjuvant chemoradiation followed by orthotopic liver transplantation				
